# Clinical and Pathological Reports of Cases of Insanity

**Published:** 1884-03

**Authors:** S. V. Clevenger

**Affiliations:** Special Pathologist Cook County Insane Asylum


### ARTICLE II.

### CLINICAL AND PATHOLOGICAL REPORTS OF CASES OF INSANITY.

By S. V. CLEVENGER, M.D., Special Pathologist Cook County Insane Asylum.

Female case No. 294. Mania in puerpero. Mary C.; age 32; Canadian; married; admitted July 26, 1883. Two weeks before, placenta prævia and difficult forceps delivery. Four days afterwards, became suddenly insane, and during a remission of furor was brought here helpless, incoherent, restless. Talked of electricity all about her; had a succession of furibund attacks; tried to walk up the wall; tore clothing; sang loudly, but seemed to recognize persons and be conscious of surroundings. Under Dr. Spray's constant care the attacks gradually lessened in severity, and the intervals grew longer between them. At times when disappointed at not seeing her husband, would become very much depressed and furor would follow, whereupon he was advised to visit her ofteher. By the last of August patient was fully recovered, and was taken home September 9, 1883.

Female case No. 223. Melancholia. Ulrica H., aged 18, German, domestic, single; admitted March 8, 1883. Would do but little else than pray continuously. Badly nourished. A maternal half sister became insane at age of 16. Mother died of variola. Father living and well. June 20 a severe, uncontrollable dysentery began, and a few hours before her death, June 26, her mind was restored, and she talked rationally, expressed a fear of death, but soon became reconciled to its approach.

Autopsy next day. Endocolitis. Body emaciated. No osseous dural adhesions. Entire brain abnormally pale. Cerebromeningeal adhesion over center of middle temporal convolution right side. Meningeal vessels full. Cerebral vessels more apparent, owing to paleness of brain. No asymmetry. An arachnoid cyst, size of a walnut at center of cerebellar base, containing clear fluid. As in melancholia there are rarely to be found brain lesions of any kind, it would be well to attach but little importance to this cyst in any ætiological sense. However, it is *possible* there may have been a connection between it and the reduction it may have undergone through the colliquative discharges preceding her restoration of reason. The etiolation of the brain would, however, point to a depressed quantitative or qualitative blood condition as more to be regarded in her case.

Female case No. 849. Typhomania. Adelaide G., aged 24, German seamstress, single, admitted Sept. 27, 1883. Two months previously, arrived in Chicago from interior of State; alleged that an expressman had outraged her next day, since when she had wandered about the streets distractedly. Two front teeth missing, sordes abundant, mouth parched, general emaciation. Erythematous patches on legs, which, if nodosum, must have been in recovering stage, as the surface was even. Struggles against all attention, raves and presents other evidences of acute delirious mania; constantly repeats "Ich bin Gott's Kind." Medicine administered with difficulty. Insomnolence extreme. Nov. 15, less delirious, but still furibund. Nov. 30, fever subsided but refuses to speak much, acts sillily, is quiet, and industriously crochets all day. December, stands by window gazing out vacantly, sometimes weeps. January, answers in monosyllables, laughs childishly if addressed. February, the expression of fatuity common in terminal dementia more apparent. Is more inclined to idleness and apathy.

Female case, No. 310. Monomania. Ellen Z., aged 26. Stockholm, clerk, single, was "queer" since seven years old. Past four weeks before admission, imagined herself a Princess of Sweden. Admitted August 16, 1883. Is very quiet and dignified. Can be trusted out of ward. She has a fair education, and by exercising patience her story was extracted from her disjointedly, and with digressions and ramblings among non-essentials, by which she sets great store. She suddenly came to the conclusion that she was a Swedish princess, because in Stockholm the members of the family she was raised with were unlike her in tastes, aspirations, etc., and certain persons had been very kind to her in America, a condescension she construed into a recognition of superiority. Things she read in newspapers and various insignificant matters appeared to her mind confirmation of her dominant idea. This is an exaggerated phase of the suspiciousness Shakespeare decribes as causing jealousy to see justification in trifles. Both the sane and the insane, when ridden by an idea, see everything in unusual ways. The patient wrote long letters to the superintendent, offering him $40,000 for her release, stating that as she intended to spend $25,000 for a musical education suitable to her royal position, time was a consideration to her. With these grandiose, systematized, reasoned-out delusions, she had the delusions of persecution natural to the disorder from the opposition she met with everywhere. She grew gradually less obtrusive with her delusions, and by October confessed to me that there might be nothing in them ; a little later asked seriously if she really were crazy, and had been imagining all these matters; Nov. 19, 1883, she was discharged, having yielded up all her false notions and apparently fully recovered.

The fact of her peculiarities being noticeable when quite young, and augmenting suddenly as she matured, would show how imbecility and monomania may be graded into one another. Spitzka regards them as allied. Had these delusions appeared earlier she might have been classed as a hebephreniac, though with decidedly monomaniacal tendencies.

Female case No. 70. Terminal dementia. Apaline H., aged 37, German, married; admitted June 16, 1877. Insane eight years before admission. Entered on old books as maniacal. Had been a hard working woman, with a worthless husband and eight children. It is said her insanity dates from burning of her property. She has passed to the demented stage of filthiness, incoherence, and unconcern for everything but eating. Both a son and daughter are also in the asylum. With the proof of heredity in their cases, their ages would indicate the mother's predisposition to insanity dated to her juvenility, and renders it probable that she inherited the taint.

Female case No. 305 (daughter of prèceding female case, No. 70). Hebephrenia. Nellie H., aged 20, American, single; admitted August 9, 1883. Insanity has been approaching gradually "for years." Depressed, with occasional silliness and explosions of anger. Is in fair health; careful to dress neatly; converses fairly well. She seemed to realize her condition, and on arrival begged not to be taken to the same ward where her mother was. She asked anxiously daily if we did not consider her as better and appearing much improved. She was sent home August 26, but her friends returned her in a nearly demented state November 24 following. Deterioration is gradual, but sure. Her face is growing "soggy," expressionless, and she often stands in one place for hours unconcerned in her surroundings.

Male case No. 311 (son of preceding female case, No. 70). Hebephrenia. Edward H., aged 25, American, single; admitted October 25, 1883. His insanity has gradually approached during past years. Three weeks before admission began to chase peddlers; became noisy and more silly than formerly. Imagines himself a big Indian. The following is from his pen, and a fair sample of insane literature:

"Cook Co Insane Asylum Cook Co Ills "Dear ——: I am still in the insane Asylum getting along well and if You have anything to send me Please bring it Yourself or send it by mail Yours ever lovingly my mother is getting better I hope ten Years is a long time for a lady to be out of her mind. I ask Your mother for Your hand She as You may remember concented my fortune is small but my love is big, and my hate is still bigger so remember if You have anything to send to me Geog w washington Stone Dead or alive. You can do as you wish I lost my cent my Post office money orders have been taking from me and Whatever wase done with them I dont know. They may have locked them up taken away from me and Yoused for some other purpose You can speak to your mother or write and ask Your Father to Please and help you if he can and then some time tell him I asck You for your hand as my WIFE and in case of war I must leave You at home, unless you are willing to follow me, but I would rather leave you at home with your mother in such times so I will be more ably to attend to my bussiness better when I am alone thats the reason Dear —— when I tend to bussiness I allwase go alone and let me know if Fred —— and your mother hase asck your pardon for what they done to you and me He Insulted me and You in my absent tell your Father how you fell agaist me for protecktion My Mother and my Fathers left wills for me but I never see them, or, they were never shone to me after me visiting Chicago three different times so I think it would be nessesery to have my Parents attend to this matter of bussiness, for me, or if they whant me to attend to it for them I can do so but would rather my *Father* would attend to it for me Chief Apach and several other tripes have been wronged and they or rather I came to them I smoked the pipe with them and the pipe said War, my tripe are the Commanches they said pease. when I smoke pipe it meant pipe, and when we whant Peasee wee make our own pease or our own warr) Ingland whants war let them make there own warr my Country lives in pease unles they force use and thats what they have done. so let them learn to make pease with Americka. My mother named me when first born Washington

Yours truly Edward"

"P. S. Notice this letter well and save it for me to read again when I return home or return to Chicago as I have no home, only among my tripe of Indians the Comanch."

The systematized delusion does not make him a monomaniac, as some would suppose, for he does not attempt to reason about it.

Female case No. 136. Recurrent Insanity. Ellen D., aged 36, Irish, married, admitted June 4, 1881. In 1879, two months before her fifth baby was born, the house in which she lived burned down, and she was exhausted in the endeavor to save property. Rallied a little till the baby was five months old, when headaches and wakings at night, imagining house on fire, began. On the streets, soon after, was seized with blindness and vertigo, followed by overpowering desire to return to Ireland. Sold off household goods without husband's knowledge and became indifferent to children; was in asylum five months and discharged apparently recovered. Headaches continued with frightful dreams, increasing with pregnancy. Six weeks before last confinement, came to Chicago from St. Louis, became depressed, and fearing to be sick without shelter went to the County Hospital, thence to poorhouse, where child was born the day following. Mania in puerpero began, and she was brought here. Husband said to be a drunkard and abusive to her. During long intervals she would be perfectly rational, industrious, and quiet. She is naturally well behaved and gentle, but two or three times yearly she would become morose and furibund, swear and use obscene language, for a week or more, and then recovering become very penitential, apologizing to all, saying she remembered everything but was powerless to prevent her bad behavior.

Last attack was in February, 1883; in July some of the usual prodromata appeared, which she noticed as preceding her trouble, such as pain in side and back, with tongue furred and bowels costive. Slight purgation caused all these to disappear. Apparently recovering.

Female case No. 137. Terminal Dementia. Mary H., aged 26, Irish, single, domestic, admitted June 9, 1881. Facial erysipelas, relatives state, "lasted two years preceding her insanity," which began with furor and verbigeration, and at this writing has progressed to the chattering incoherency of dementia, without its abject helplessness. She works in the asylum dining-room quite willingly, and is much better off while so engaged. She is very abusive and talkative, mocking everyone, but not destructive. Appears to be chronically angry, but is satisfied with incessant scolding. No hereditary taint. She occasionally makes demonstrations with butcher knives, and under bad management would do harm, but a piece of cake or bread, or the least kindness tendered her, quickly changes the current of her thoughts and intentions.

Female case No. 50. Imbecility. Keziah S., age 44, American, single, admitted Feb. 24, 1876. Reads and writes a little. Dangerous if unrestrained; uncontrollable temper when at home; thinks everyone steals from her; incoherent, abusive, and at times very noisy; dislikes men; loves to annoy everyone; microcephalic; never known to be sick. Mother died of some stomach trouble; father of apoplexy, at age of 80. Mother just previous to patient's birth attended grandfather in last illness, and was overworked. Menstruates regularly. Rachitic in infancy. She is so suspicious that others will steal from her that she insists upon washing all her own clothing; will not trust it in laundry. If anyone touches anything belonging to her she keeps up an incessant chatter, half shriek and half gabble.

Female case No. 3. Terminal Dementia. Catherine L. age 32, American, single, admitted Jan. 1, 1866, but was in insane department of poor-house in 1857. She is called "Katy Darling," possibly from having sung that song years before demented stage reached. Was originally destructive with frequent furor maniacorum. Has a sister here also insane, (case No. 92). Mother died with pneumonia, aged 50. Father had bad reputation; deserted his family and gave mother much trouble during pregnancy, which is claimed by others in family as predisposing cause of insanity. Exciting cause, disappointment in love affair. "Katy" works faithfully in ward dining room, seems happy, but is incoherent, and has an expressionless face. Assumes much importance, and insists upon patients keeping order in her domain, in which she is very helpful.

Female case No. 92. Terminal dementia. Mary F., age 35, American; married; admitted May 23, 1879, (sister to case No. 3); was always nervous and inclined to melancholia. Insanity first noticed in 1872; husband left home and business and she worried accordingly; Nov. 1877, miscarried, after which developed melancholia agitata, which passed rapidly to terminal dementia. At first she afforded the sentimental idea of insanity, crouched upon the floor with her beautiful black hair drawn across her face, passing her fingers listlessly through the meshes, but while her condition is more nearly that of her sister's, taking some interest in her surroundings, her bloated visage and fleshiness, recently acquired, reveal the hopelessness of her final stage.

Female case No. 38. Mania. Unknown name; age 59; nothing can be ascertained concerning her; she persists in calling herself Mrs. Lincoln; says she killed "old Abe," her husband; lies inordinately about everything; is obscene, and upon slight provocation furious; fights and swears readily. When asked what her name was before she was married, says it was Todd, thus being consistent in her delusion.

Female case No. 40. Hypomania. Ingar R., aged 46; Norwegian; married. Is quite useful in her ward, helps with the sewing and cleaning, but constantly mutters her orders to everyone. She imagines herself a queen; is not certain as to what kind of a queen she is; readily assents if told she is Queen of Sweden, Queen Victoria or Queen of Tragedy. Her ideas of regal authority are mixed with that pertaining to a police magistrate; she fines anyone five dollars for smoking in her presence; frowns severely upon all, but smiles complacently if petted and flattered. She is not a monomaniac, because her ideas are unsystematized, and no attempt at reasoning is made; nor is she a simple maniac, because with the exaltation of feelings she has no furor at any time. She dresses on state occasions with tinsel crown, and any gaudy trumpery she can find. She has hallucinations of hearing and sight, but of a pleasurable kind.

Female case No. 14. Mania. Wilhelmina S., aged 57, German; admitted January 6, 1871. Chronically verbigerative, abusive, noisy, vile talker. Has hallucinations of sight and hearing. Dislikes and attacks everyone except Dr. Spray, whom she imagines is her son. She has covered the walls of her room with rude drawings and appropriately silly inscriptions. Her day's work consists in standing at the window swearing at passers-by, bobbing her head to and fro with rage. It is amusing to see how her manners change as soon as her "son" appears.

Female case No. 127. Katatonia. Ellen P., aged 23, American, single; admitted in 1880. One of the most interesting cases in the asylum. Cataleptoidal and stuporous usually three months at a time, during which saliva oozes from her mouth, and she maintains for hours any position in which she may be placed, however awkward. Arms may be elevated, and they will remain as placed. Has the waxy mobility of this disorder marked. Suddenly emerges from stupor, becomes lively and tidy, and invades rooms of other patients, turns repeated somersaults up and down the corridor. Melancholia follows this stage, and lasts about three months. Catalepsy lasts longer in winter than in summer months. Never has menstruated, to attendant's knowledge. Since childhood she has been peculiar in looks and manner, and at age of 14 was more insane, and taken to Batavia asylum and thence home, but her troublesome pranks compelled her being brought here. Last cataleptoidal state began in December, 1883, lasting till January 31, 1884, wheu she arose in the morning, made her bed, and was quite lively and garrulous, but relapsed later in day. Next day the same thing occurred, but stupor not so marked. February 3, 1884, maniacal stage fully developed, and continues.

Male case No. 5. Mania. Dietrich S.; admitted July 14, 1869. Present age 59; German. First attack sixteen years duration. Spends nearly his entire waking time hammering on the head of a nail. He says every time he hits it he strikes the devil on the head.

Has two fistulous openings in left leg, fibular side below knee, made by bullet wounds at battle of Vicksburg, while a soldier in the late war. He keeps the wounds irritated by pieces of lint inserted in them, as while discharging his health and mind are better. Mania developed after the battle, but "was crazy a little before the war."

Father died of "kidney disease," aged 54; mother of phthisis pulmonalis, aged 57.

Male case No. 60. Monomania. Samuel N., admitted January 30, 1879; aged 54, English, lithographer, spiritualist. Three years insane prior to admission. Says he was arrested for writing an article in the *Religio-Philosophical Journal*, at the request of the editors, who, he claims, are afraid of him. Not intemperate. Talks very rationally. Worked in drug store of asylum four years; jocularly remarked that he "never got further than pounding cinchona bark." Recently at work in asylum store-room. Is very industrious, and can be trusted anywhere on any errand, but every morning he adorns the trees and public spots around the building with proclamations against spirits, mediums and ghosts. His fulminations are embellished with off-hand geometrical figures, doubtless suggested by his lithographic career, but these figures evidently serve some cabalistic purpose. Here is one of his least offensive documents. It is headed with a sketch of a church: "Little Church Round the Corner. Moral Church we bury our brothers in one peice In honor of the cannons of our order Ladi Lado Lade Ladum Lady These ladies know nothing about Red Stocking In Honor of the Nitric Acid Ceremonies." Which smacks more of freemasonry than another one, wherein he inveighs against certain bastard mediums, and threatens to take away their spiritualistic control. I have a collection of his pronunciamentos.

Male case No. 24. Monomania. Admitted March 18, 1873. James C., age 45, Irish, lawyer, married. Verbigerative. Delusions systematized. This is one of our "show patients." He has a very plausible delivery, and tells you confidentially how a certain judge before whom he plead turned into a boa-constrictor, and the jury into devils. Satan bothers him considerably. July, 1883. He is undergoing more metamorphoses than Ovid dreamed of. He used to wipe one eye with a moist salted rag until the optic was quite inflamed. Now he says he has cast off thirteen skins. Employs his entire time blowing snakes from his nose, and expects soon to be transfigured. Much of his acquired legal adroitness clings to him. For example, a visitor, thinking it best to acquiesce in all an insane person suggested, was asked by him recently if he (the visitor) didn't know that Lincoln was President? Ans. "Yes." "Well, you know General Grant has captured Mexico?" Ans. "Yes." "And you know that Susan B. Anthony is Vice-President?" Ans. "Yes." "Well, you know a great deal more than I do, and I think you are a bigger fool." With this he turned contemptuously away.

